# Interleukin-11 promotes epithelial-mesenchymal transition in anaplastic thyroid carcinoma cells through PI3K/Akt/GSK3β signaling pathway activation

**DOI:** 10.18632/oncotarget.10831

**Published:** 2016-07-25

**Authors:** Zhaoming Zhong, Zedong Hu, Yue Jiang, Ruimei Sun, Xue Chen, Hongying Chu, Musheng Zeng, Chuanzheng Sun

**Affiliations:** ^1^ Department of Head and Neck Surgery, The Third Affiliated Hospital of Kunming Medical University, Kunming, China; ^2^ Department of Medical Oncology, The First Affiliated Hospital of Kunming Medical University, Kunming, China; ^3^ State Key Laboratory of Oncology in South China, Sun Yat-sen University Cancer Center, Guangzhou, China

**Keywords:** anaplastic thyroid carcinoma, interleukin-11, epithelial-mesenchymal transition, metastasis, hypoxia-inducible factor-1α

## Abstract

Metastasis is the major cause of treatment failure in anaplastic thyroid carcinoma (ATC) patients. In the preliminary study, we demonstrated that interleukin (IL)-11 expression is positively correlated with distant metastasis in ATC. However, the mechanisms underlying remain largely unknown. Here, we found that cobalt chloride (a hypoxia mimetic) promoted IL-11 expression via HIF-1α activation. Furthermore, the resultant increase in IL-11 expression significantly induced epithelial-mesenchymal transition (EMT) in ATC cells, accompanied by Akt/GSK3β pathway activation and increased invasive and migratory abilities. Conversely, HIF-1α or IL-11 knockdown, or treating cells with a neutralizing antibody against IL-11, a PI3K inhibitor, or Akt inhibitor V, significantly suppressed the induction of EMT and counteracted the enhancements in invasive and migratory abilities. These results indicate that hypoxia increases IL-11 secretion in ATC cells via HIF-1α induction and that IL-11 then induces EMT in these cells via the PI3K/Akt/GSK3β pathway, ultimately improving their invasive and migratory potential. This study elucidates the prometastatic role played by IL-11 in ATC metastasis and indicates it as a potential target for the treatment of cancer metastasis. However, many questions remain to be explored.

## INTRODUCTION

Anaplastic thyroid carcinoma (ATC) is one of the most aggressive malignant tumor types in humans, accounting for 1.6-5% of all thyroid cancers. However, it is responsible for 14-50% of all thyroid carcinoma-related deaths [1–8]. It was reported that high distant metastasis at presentation is the most important reason for the dismal prognosis [1, 4, 6–9]. McIver et al [10] reported that 46% of a cohort of 134 ATC patients had evidence of metastatic disease at presentation and that 68% of the patients developed metastatic disease at some stage of their illness. Multimodal therapy can achieve better results in terms of avoiding death from local invasion and suffocation [6, 11] but may have no effect on distant disease [10]. Thus, only 20% of affected patients survive for 1 year after diagnosis, and the median survival duration is 3-9 months [5, 6, 8, 9, 11]. Therefore, there is a desperate need to reveal the mechanism underlying ATC metastasis and to identify therapeutic targets.

ATC typically presents as a rapidly enlarging solid tumor, thus, the ATC cells often face serious nutrient deprivation and hypoxia. The best-characterized response to hypoxia is the induction of hypoxia-inducible factor (HIF)-1 [12, 13]. HIF-1 is composed of αβ heterodimers: the β-subunit is constitutively expressed, whereas expression of the α-subunit is tightly regulated by oxygen [12, 13]. Recent studies have shown that interleukin (IL)-11 gene expression can be induced by hypoxia and contributes to the distant metastasis of several types of solid carcinoma, including breast carcinoma, chondrosarcoma, choriocarcinoma, [14–19]. Other studies have also shown that IL-11 stimulates inflammation, as well as cancer motility and invasion, through the JAK/STAT3, PI3K/Akt, and Ras/ERK pathways, ultimately resulting in an aggressive phenotype [20–22]. However, whether IL-11 contributes to ATC metastasis and by what mechanism(s) remain unknown.

Epithelial-mesenchymal transition (EMT) is a process in which epithelial cells lose their cell-cell adhesion properties and are converted into a mesenchymal phenotype, allowing noninvasive and nonmetastatic tumor cells to acquire the capacity to infiltrate surrounding tissues and ultimately to metastasize to distant sites [23–25]. Clinical studies have revealed that EMT is closely related to tumor metastasis and poor prognosis and is considered to be the central mechanism responsible for metastasis in multiple cancers [23, 26]. Therefore, we hypothesize that IL-11 promotes ATC metastasis by inducing EMT.

In the current preliminary clinical study, we demonstrated that IL-11 expression is positively correlated with distant metastasis in ATC patients. Furthermore, we demonstrated that cobalt chloride (CoCl_2_, a hypoxia mimetic) induces HIF-1α expression, subsequently promoting the expression of IL-11. Following this, IL-11 induces ATC cells to undergo EMT via the PI3K/Akt/GSK3β signaling pathway, ultimately promoting their invasiveness and migratory abilities. This discovery could lead to the identification of possible drug targets.

## RESULTS

### IL-11 expression in paraffin-embedded ATC and PTC samples and its clinical significance

**Figure 1:**
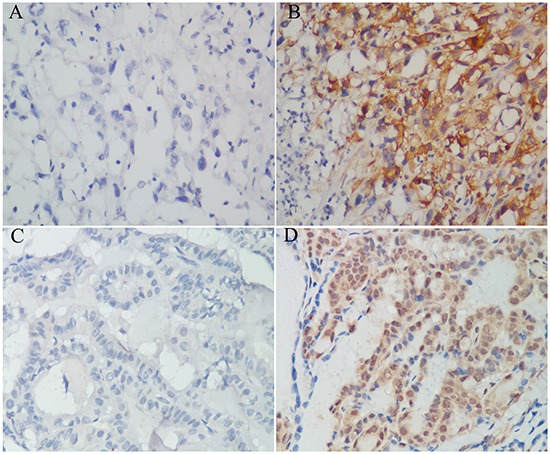
IL-11 expression in paraffin-embedded anaplastic thyroid carcinoma and papillary thyroid carcinoma specimens Negative expression **A.** and positive expression **B.** of IL-11 protein in anaplastic thyroid carcinoma tissues. Negative expression **C.** and positive expression **D.** of IL-11 protein in papillary thyroid carcinoma tissues.Original magnification is 400 ×.

To assess the clinical significance of IL-11 in our patient cohort, we examined IL-11 expression in 76 paraffin-embedded ATC samples by immunohistochemistry. As a result, 22 cases (28.9%) showed negative IL-11 expression, and 54 (71.1%) cases showed positive IL-11 expression (Figure 1A-1B). In association analysis, positive IL-11 expression was associated with distant metastasis and advanced cTNM stage (Table 1). As a control, 46 (46.0%) cases showed positive IL-11 expression in paraffin-embedded PTC tissues (Figure 1C-1D), which was significantly lower than that for the ATC tissues (*χ*^2^ = 11.046, *P* = 0.001).

**Table 1:** Correlations between IL-11 expression and clinical features in 76 patients with ATC

Item	n	Deaths (n)	IL-11		*χ*^2^ value	*P*
			Negative	Positive		
Sex						
Men	38	33	12	26	0.064	0.800
Women	38	35	10	28		
Age						
<60 years	36	29	12	24	0.299	0.585
≥ 60 years	40	39	10	30		
Primary tumor size						
< 6 cm	45	39	14	31	0.059	0.807
≥ 6 cm	31	29	8	23		
Lymph node metastasis						
N0	27	25	7	20	0.028	0.867
N1	49	43	15	34		
Distant metastasis						
M0	57	49	20	37	4.180	0.041
M1	19	19	2	17		
Stage (AJCC, 2010)						
IVA	5	1	4	1	9.555	0.008
IVB	52	48	16	36		
IVC	19	19	2	17		

Abbreviations: IL-11, interleukin-11; ATC, anaplastic thyroid carcinoma; AJCC, American Joint Committee on Cancer

### ATC cell lines exhibit increased IL-11 expression

**Figure 2:**
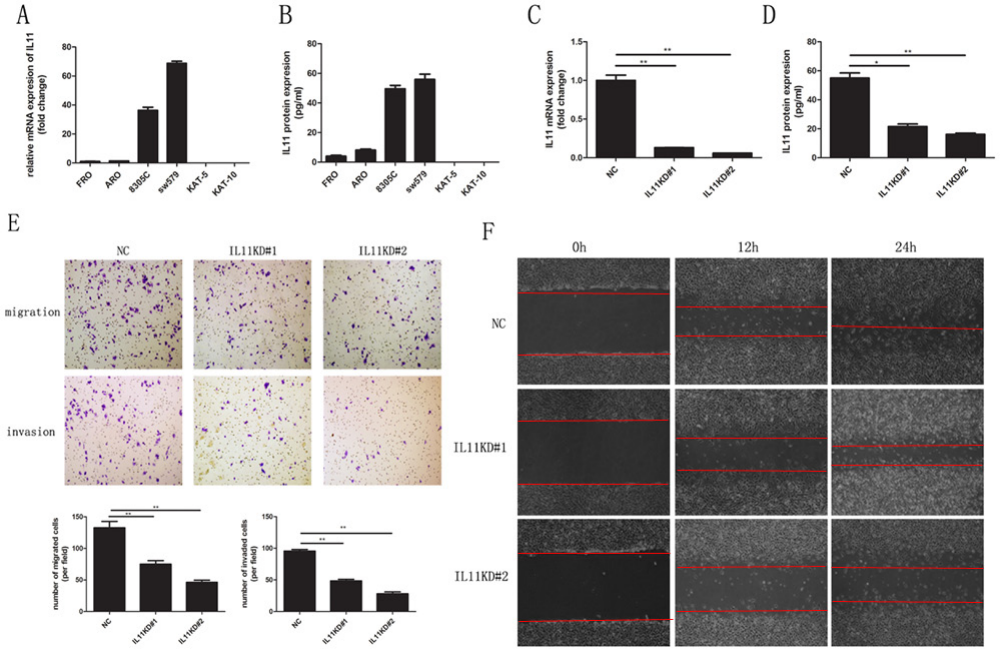
IL-11 promotes invasive and migratory potential in ATC cells IL-11 mRNA **A.** and protein **B.** levels are significantly higher in ATC cell lines (ARO, FRO, 8305C, and sw579) than in differentiated thyroid carcinoma cell lines (KAT-5, KAT-10), as measured by quantitative RT-PCR and ELISA, respectively. After knocking down IL-11, the mRNA **C.** and protein **D.** levels of IL-11 in sw579 cells significantly decreased, as measured by quantitative RT-PCR and ELISA, respectively. After knocking down IL-11, the invasive and migratory abilities of sw579 cells significantly decreased, as assessed by transwell assay **E.** and wound-healing assay **F.** *P < 0.05, **P < 0.01, ***P < 0.001 relative to the controls (Student's t-test). Photomicrographs are at 100× magnification.

To measure IL-11 expression in thyroid cancer cell lines, quantitative RT-PCR and ELISA were conducted on ATC (FRO, ARO, 8305C, and sw579) and differentiated thyroid carcinoma (DTC; KAT-5 and KAT-10) cell lines. Quantitative RT-PCR revealed higher IL-11 mRNA expression in the ATC cell lines compared with the DTC cell lines. In fact, there was almost no IL-11 mRNA expression in the DTC cell lines (Figure 2A), and similar results were found for IL-11 protein expression based on ELISA (Figure 2B). Thus, our data indicated that IL-11 expression significantly increased at both the mRNA and protein levels in the ATC cell lines.

### IL-11 promotes the invasive and migratory abilities of ATC cells

**Figure 3:**
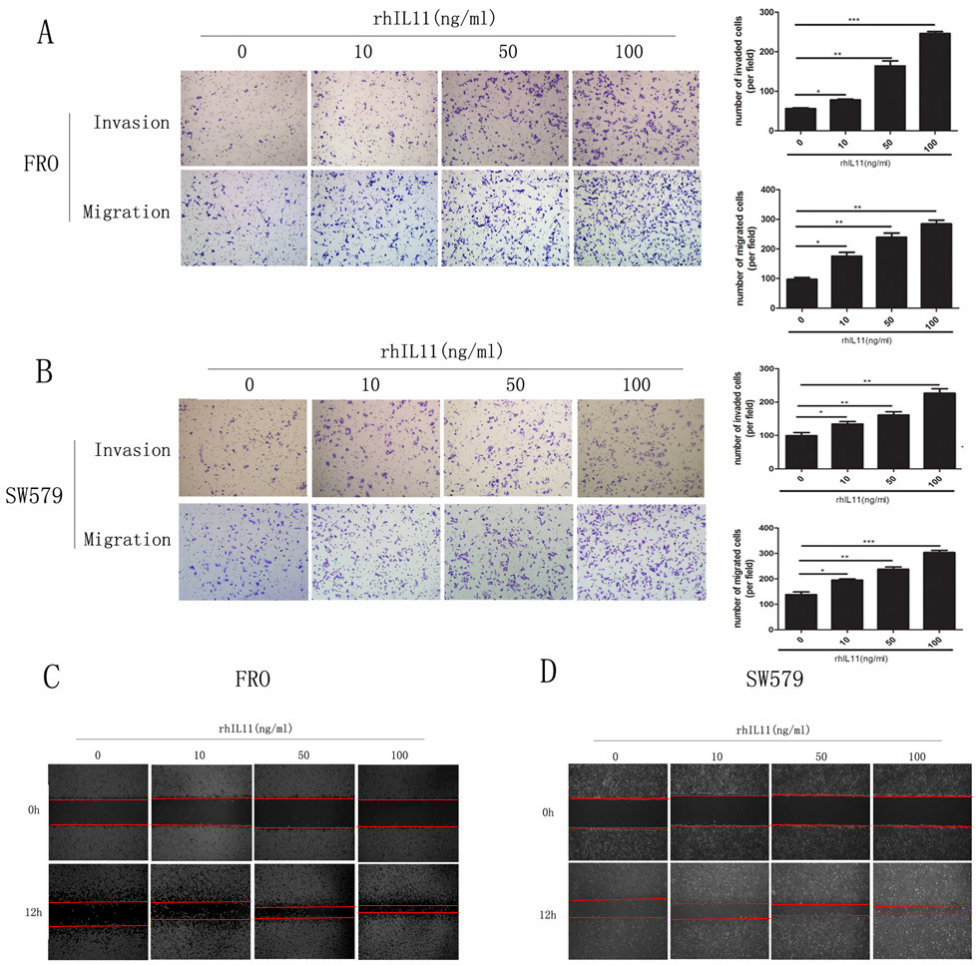
Exogenous IL-11 significantly enhances the invasive and migratory abilities of ATC cells **A, B.** Transwell assays for FRO and sw579 cells in the presence or absence of rhIL-11 at increasing concentrations (10–100 ng/ml). Migrated cells were plotted as the average number of cells per field. *P < 0.05, **P < 0.01, ***P < 0.001 relative to the controls (Student's t-test). Photomicrographs are at 100× magnification. **C, D.** Wound-healing assays using FRO or sw579 cells treated with rhIL-11 at increasing concentrations (0–100 ng/ml).

To clarify IL-11′s role in ATC cell invasion and migration, we generated sw579 cells with stably knocked down IL-11 expression using retroviral vectors (IL-11KD#1 and IL-11KD#2); scrambled shRNA was used as a negative control (NC). Quantitative RT-PCR and ELISA were performed to assess IL-11 knockdown efficiency. As seen in Figure 2C and 2D, IL-11 mRNA and protein levels were significantly lower in IL-11KD#1 and IL-11KD#2 cells than in NC cells, confirming the knockdown of IL-11 in these cells. Invasion and migration assay results revealed that suppressing endogenous IL-11 markedly reduced the invasive and migratory capacities of sw579 cells (Figure 2E). Wound-healing assay results also indicated that IL-11 shRNA-transfected sw579 cells displayed decreased migration compared with NC cells (Figure 2F). However, IL-11 knockdown did not significantly suppress the proliferation of sw579 cells, as shown in the results of proliferation and colony formation assays (Supplementary Figure 1A and 1B). Furthermore, we treated ATC cells with exogenous rhIL-11 and found that their invasive and migratory potential significantly increased in a concentration-dependent manner (Figure 3A and 3B). Similarly, the wound-healing assay results after using rhIL-11 to treat ATC cells were the same as those produced following IL-11 shRNA-mediated knockdown (Figure 3C and 3D). Taken together, these results suggest that IL-11 promotes ATC cell invasion and migration.

### IL-11 induces EMT via the PI3K/Akt/GSK3β pathway

**Figure 4:**
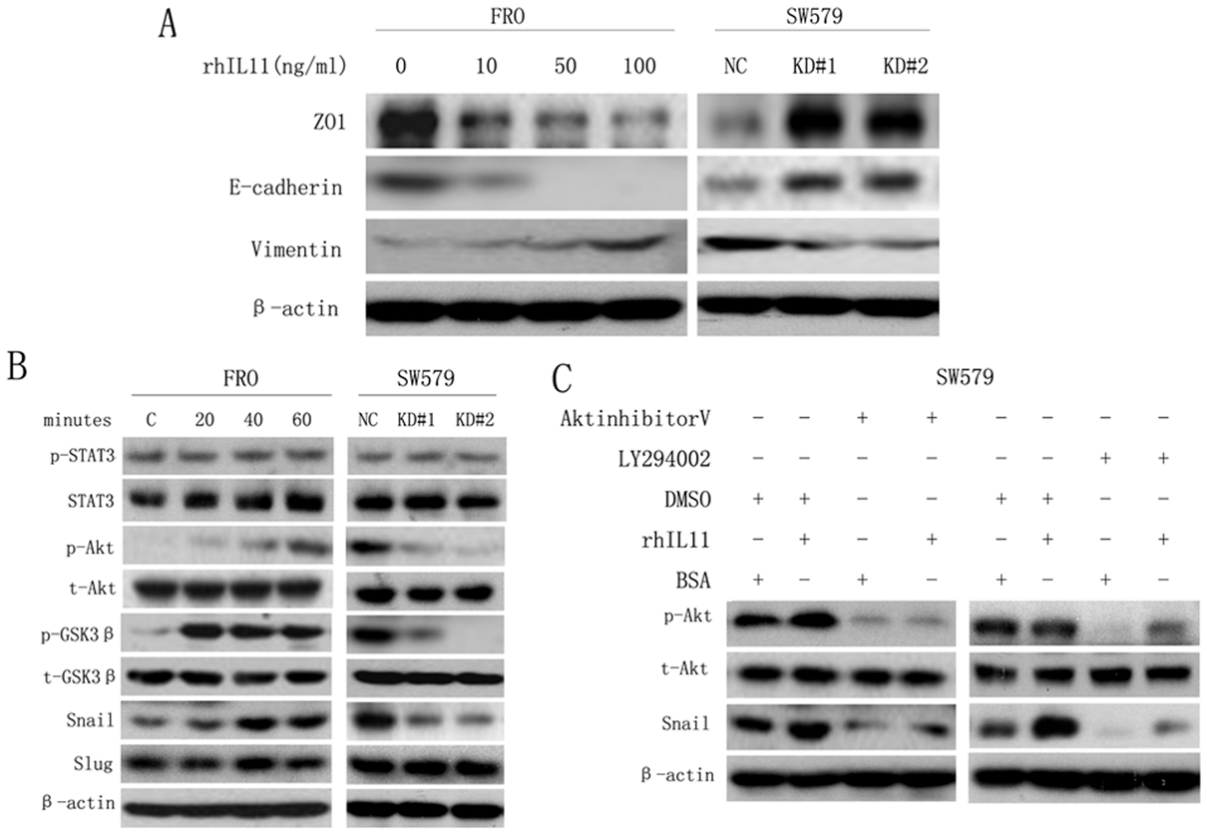
IL-11 induces EMT in ATC cells via the PI3K/Akt/GSK3β signaling pathway **A.** Western blotting to detect the expression of EMT markers in FRO cells treated with rhIL-11 at increasing concentrations (0–100 ng/ml) and in sw579 cells expressing either IL-11 shRNA or the NC. **B.** Western blotting to detect EMT and Akt/GSK3β pathway markers in FRO cells treated with rhIL-11 for increasing amounts of time (0–60 minutes) and in sw579 cells expressing either IL-11 shRNA or NC. **C.** Western blotting to detect EMT and Akt/GSK3β pathway markers in sw579 cells after pretreatment with 20 μM Akt inhibitor V or LY294002 or 100 ng/ml rhIL-11 for 60 min. β-Actin was used as a loading control.

EMT is a central mechanism underlying metastasis in various cancers [23–25]. To assess whether IL-11 induces EMT in ATC cells, we treated ATC cell lines with increasing concentrations of rhIL-11 (0–100 ng/ml) and then analyzed epithelial and mesenchymal markers by western blotting. As shown in Figure 4A, the cells exhibited a typical EMT phenotype in a concentration-dependent manner, including down-regulation of the epithelial markers E-cadherin and ZO1 and up-regulation of the mesenchymal marker vimentin. However, IL-11 knockdown reversed the EMT phenotype. As PI3K/Akt pathway activation is emerging as a central component of EMT [25, 27], we inferred that IL-11 induces EMT in ATC cells via PI3K/Akt signaling. As shown in Figure 4B, rhIL-11 induced Akt (Ser 473) phosphorylation in FRO cells in a time-dependent manner, whereas Akt phosphorylation was inhibited in IL-11KD#1 and IL-11KD#2 cells. Furthermore, Akt phosphorylation was accompanied by the activation of GSK3β, a downstream target protein of Akt. These results suggested that IL-11 may induce EMT in ATC cells via the Akt/GSK3β pathway. As a member of the IL-6 family, IL-11 may be related to STAT3 pathway activation. However, neither rhIL-11 treatment nor IL-11 knockdown changed the phosphorylation level of STAT3 in the present study (Figure 4B).

One EMT hallmark is the loss of E-cadherin expression, which is negatively regulated by the transcription factors Snail and Slug [23, 8–31]. Therefore, we examined Snail and Slug expression in ATC cells treated with rhIL-11. As a result, we found that Snail expression significantly increased in a time-dependent manner but decreased in IL-11-knockdown ATC cells, whereas no significant changes in Slug expression were observed (Figure 4B). Because GSK3β plays an important role in the maintenance of epithelial architecture by regulating Snail [27, 32], we used the PI3K inhibitor LY294002 and Akt inhibitor V to investigate whether the upregulation of Snail was caused by PI3K/Akt/GSK3β pathway activation in sw579 cells. Treatment with LY294002 or Akt inhibitor V significantly reduced the levels of phosphorylated Akt and Snail (Figure 4C). Similarly, the enhanced invasive and migratory abilities of ATC cells induced by rhIL-11 were significantly suppressed by LY294002 (Supplementary Figure 2A). These findings suggested that IL-11 induces EMT and promotes invasion and migration in ATC cells via the PI3K/Akt/GSK3β/Snail pathway.

### Cobalt chloride promotes IL-11 expression in ATC cells via HIF-1α

**Figure 5:**
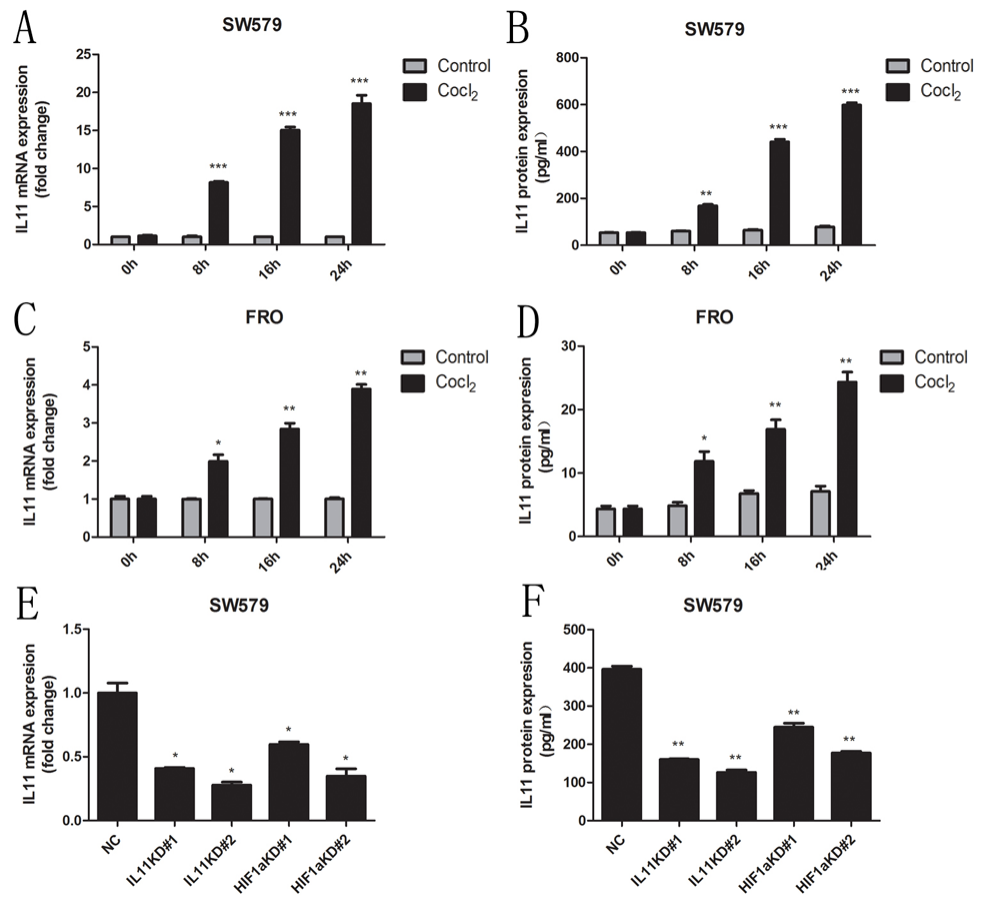
CoCl_2_ induces IL-11 secretion Relative mRNA levels of IL-11 in sw579 **A.** and FRO **C.** cells induced by 0.1 mM CoCl_2_ at the indicated times, as measured by quantitative RT-PCR. IL-11 concentrations in sw579 **B.** and FRO **D.** cells following treatment with 0.1 mM CoCl_2_ at the indicated time, as measured by ELISA. Relative mRNA levels **E.** or protein concentrations of IL-11 **F.** in sw579 NC-, IL-11 shRNA- and HIF-1α shRNA-transfected cells, as measured by quantitative RT-PCR and ELISA.

As IL-11 was recently reported to be a hypoxia-inducible gene [19], we treated ATC cells with 0.1 mM CoCl_2_ (a hypoxia mimetic). Treatment with CoCl_2_ significantly increased IL-11 production in a time-dependent manner (Figure 5A-5D). Because sw579 cells exhibited high IL-11 expression following treatment with CoCl_2_, this cell line was chosen for further investigation. As HIF-1α expression is tightly regulated by oxygen, we used a retroviral vector expressing two distinct shRNA sequences targeting HIF-1α (HIF-1α KD#1 and HIF-1α KD#2) to transfect sw579 cells; scrambled shRNA was used as a negative control (NC). After treating IL-11 shRNA- and HIF-1α shRNA-transfected sw579 cells with 0.1 mM CoCl_2_, we found significantly decreased IL-11 expression in both cell lines compared with NC cells (Figure 5E and 5F). These findings suggested that CoCl_2_ promotes IL-11 expression in ATC cells via HIF-1α induction.

### IL-11 promotes hypoxia-induced EMT in ATC cells via HIF-1α

**Figure 6:**
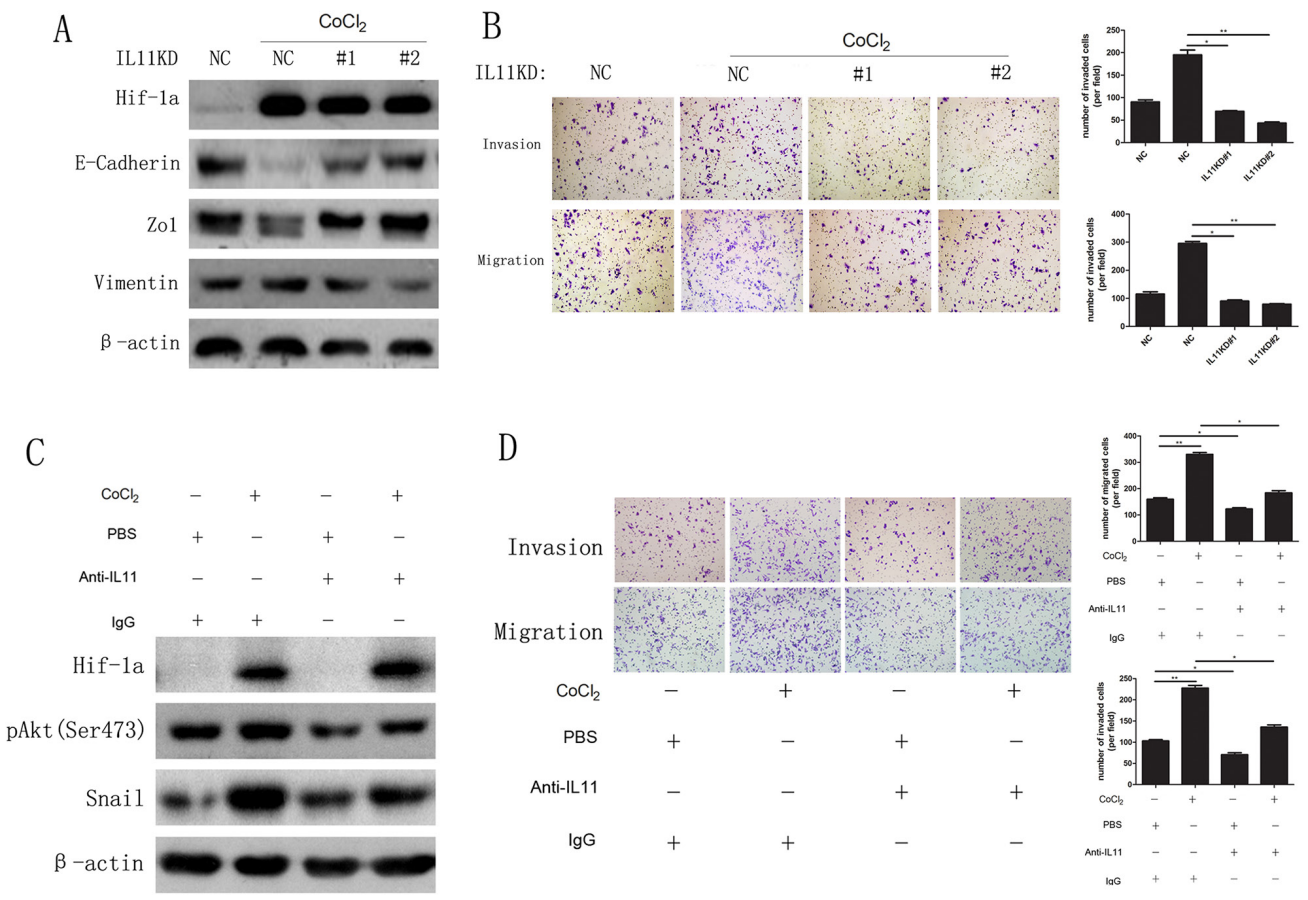
CoCl_2_ promotes EMT by inducing IL-11 secretion **A.** Western blotting of IL-11 shRNA- or NC-transfected sw579 cells after treatment with 0.1 mM CoCl_2_ for 24 hours using the indicated antibodies. **B.** The invasive and migratory abilities of IL-11 shRNA- or NC-transfected sw579 cells with or without 0.1 mM CoCl_2_ exposure, evaluated by transwell assay. **C.** Western blotting for phosphorylated Akt and Snail in sw579 cells treated with or without 0.25 μg/ml anti-IL-11 antibody or 0.1 mM CoCl_2_. β-Actin was used as the loading control. **D.** The invasive and migratory abilities of IL-11 shRNA- or NC-transfected sw579 cells treated with or without 0.25 μg/ml anti-IL-11 antibody or 0.1 mM CoCl_2_, evaluated by transwell assay. Migrated cells were plotted as the average number of cells per field. Photomicrographs are at 100× magnification. Bars correspond to the mean ± SD.

As hypoxia can promote EMT in many cancer cell types via HIF-1α induction, we treated FRO and sw579 cells with 0.1 mM CoCl_2_ or 1 mM dimethyloxalylglycine (DMOG, a HIF-1α degradation inhibitor). The results clearly revealed that both FRO and sw579 cells underwent EMT (Supplementary Figure 3A) concomitant with significant increase in IL-11 mRNA levels relative to those of untreated cells (Supplementary Figure 3B). To investigate the role of IL-11 in HIF-1α-induced EMT, we treated IL-11 shRNA-transfected sw579 cells with CoCl_2_ (0.1 mM). We found that IL-11 knockdown did not change HIF-1α expression, whereas it significantly increased E-cadherin and ZO1 expression and decreased vimentin expression compared with NC cells (Figure 6A), implying that IL-11 knockdown significantly suppresses EMT in sw579 cells. On the other hand, we found that cells with IL-11 knockdown exhibited significantly decreased invasive and migratory capacity compared with NC cells treated with CoCl_2_ (Figure 6B). Finally, we used an anti-IL-11 antibody (0.25 ng/ml) to neutralize the effect of IL-11 secretion on sw579 cells. As shown in Figure 6C, the levels of phosphorylated Akt and Snail in sw579 cells significantly decreased. Accordingly, treatment with the anti-IL11 antibody reversed the enhanced invasion and migration of ATC cells induced by 0.1 mM CoCl_2_ (Figure 6D). These results demonstrated that IL-11 promotes hypoxia-induced EMT and increases ATC cell invasion and migration via HIF-1α induction.

## DISCUSSION

Metastasis is the leading cause of death in ATC patients. Close to 50% of ATC patients have distant metastases at presentation, indicating that ATC cells have vigorous invasive and migratory capacities. Thus, uncovering the mechanism of ATC metastasis is of key significance in decreasing the death rate of ATC patients. However, few studies have focused on how ATC cells acquire metastatic potential. Recently, Yun et al [8] used immunohistochemistry to investigate the expression of various cancer stem cell markers in a panel of thyroid carcinoma tissues, including 133 papillary thyroid carcinomas and 25 ATC specimens. The authors found a complete absence of CXCR4 expression in papillary thyroid carcinoma, whereas at least focal CXCR4 expression was found in ATC. Furthermore, high CXCR4 expression was closely correlated with distant metastasis; however, the manner in which CXCR4 promoted metastatic potential in ATC was not revealed.

Epithelial-mesenchymal transition, a multi-step process involving molecular and cellular changes in epithelial cells, is an essential step toward successful metastatic spread [33]. These changes are governed by transcription factors, including Twist1, Snail, Slug, and Sip1 [33]. To date, a number of different EMT inducers, such as Wnt, EGF and TGF-β, have been identified, and numerous molecular pathways have been delineated [24, 33–35]. Tissue hypoxia is a common microenvironment for solid tumors with rapid growth. Recently, Onnis was the first to demonstrate that IL-11 is a hypoxia-induced gene and that HIF-1 synergistically interacts with AP-1 to activate the IL-11 promoter [19]. Our current study showed that ATC cells secrete IL-11 in a time-dependent manner under conditions that mimic hypoxia (treatment with CoCl_2_), and knocking down HIF-1α expression significantly decreases the induction of IL-11 secretion by these conditions. During this process, mimicking hypoxia promotes EMT and enhances ATC cell invasion and migration, and knockdown of IL-11 expression or use of an anti-IL-11 neutralizing antibody significantly decreases CoCl_2_-mediated EMT induction. EMT induction in ATC cells by CoCl_2_ is accompanied by the activation of Akt and GSK3β. Both the PI3K inhibitor LY294002 and Akt inhibitor V significantly suppress EMT, invasion and migration in ATC cells. These results indicate that hypoxic conditions increase IL-11 secretion in ATC cells and that IL-11 induces EMT in these cells via the PI3K/Akt/GSK3β signaling pathway, ultimately improving their invasive and migratory potential.

Our data suggest the important role of IL-11 in promoting the metastatic potential of ATC cells is consistent with the findings of many previous studies [36–39]. Calon et al [36] reported that endogenous IL-11 increased colorectal cancer metastasis. However, IL-11 is a megakaryopoietic cytokine that stimulates platelet production, and it has been shown that recombinant IL-11 is an efficient support therapy in patients with malignancies who develop thrombocytopenia as a side effect of chemotherapy. Therefore, the prometastatic effect of IL-11 described in the current study suggests that its use in an adjuvant setting should be reassessed [36, 40]. On the other hand, that the ATC patients with elevated platelet counts had a poorer prognosis has been reported [6]. It was reported that platelets can protect circulating tumor cells from the immune system and assist them during extravasation [41]. In addition, platelets are a rich source of TGF-beta, which has been shown to significantly promote bone metastasis of breast carcinoma [42] and liver metastasis of colorectal cancer [36]. Thus, it is possible that tumor-derived IL-11 may also promote platelet production and increase TGF-beta secretion, which may further enhance the metastatic potential of ATC cells. Consequently, it is plausible that IL-11 works through many pathways to induce a more favorable scenario for ATC cells metastasis. The 71.1% of ATCs display IL-11 expression, which was significantly higher than that for the papillary thyroid carcinoma, and may help explain the high rates of ATC metastasis.

In conclusion, to the best of our knowledge, this is the first report showing that IL-11 promotes distant metastasis in patients with ATC. The prometastatic mechanism of IL-11 may be related to hypoxia-induced stimulation of IL-11 production, which promotes ATC cell invasion, migration and EMT via the PI3K/Akt/GSK3β pathway. The current study demonstrates that IL-11 could be a potential therapeutic target for cancer metastasis. However, many related issues, such as the relationship between IL-11 and platelets as well as the role of platelets during the process of ATC metastasis, remain to be explored.

## MATERIALS AND METHODS

### Patients and clinical tissue specimens

The study was approved by the Ethics Committee of the Sun Yat-sen University Cancer Center, and written informed consent was obtained from all study subjects prior to enrollment. Seventy-six paraffin-embedded ATC samples, which were consecutively pathologically diagnosed between January 1980 and January 2014 in the Sun Yat-sen University Cancer Center, were collected for immunohistochemical analysis. These samples were obtained from 38 men and 38 women, with a mean age of 65 years (ranging from 27 to 83 years). All patients were followed up until July 2014, and the median follow-up was 6 months (range: 1-237 months). Association between IL-11 expression levels and clinical features in the patient cohort are shown in Table 1. cTNM stages were assessed according to the TNM classification of the American Joint Committee on Cancer (AJCC) [43]. For this study, 100 paraffin-embedded papillary thyroid carcinoma (PTC) tissues were used to provide contrast.

### Immunohistochemical assay (IHC)

Paraffin-embedded ATC and PTC tissue specimens were cut into 4-μm-thick sections and incubated at 60°C for 2 hours. All sections were deparaffinized with xylene and rehydrated with a gradient of ethanol to distilled water. After treatment with 3% H_2_O_2_ for 15 min to block endogenous peroxidase, the sections were submerged in EDTA antigen retrieval buffer (pH 8.0) and microwaved for antigen retrieval. Then, the sections were treated with normal goat serum for 30 min to reduce nonspecific binding and incubated with a rabbit anti-IL-11 antibody (NOVUS Biologicals, USA) overnight at 4°C (dilution of 1:250). The slides were washed with PBS plus 1:1000 Tween-20 three times and incubated with an anti-rabbit secondary antibody for 30 min at 37°C. Diaminobenzidine (DAB, Zhongshan Biological and Technical Company, Beijing, China) was used as a colorimetric reagent for protein detection. As a negative control, the antibody was replaced with normal goat serum.

Tissues were considered positive for IL-11 expression when cytoplasmic and cytomembrane staining was detected. Semiquantitative expression levels were measured by assessing the percentages and intensities of the stained tumor cells. Positive cell percentage was scored in 4 grades: 0 (0–5%), 1 (6–25%), 2 (26–50%), and 3 (51–100%). Staining intensity was classified in 4 grades: 0 (no staining), 1 (yellow), 2 (deep yellow), and 3 (brown). Scoring of both parameters was calculated after counting at least 5 fields at 400 × magnification. The points scored for the percentage of positive cells and the staining intensity were added, and each specimen was classified into 1 of 2 groups according to overall score: scores of 0-2 points were classified as negative for IL-11 expression, and scores higher than 2 points were classified as positive for IL-11 expression. All slides were independently assessed by two pathologists who were blinded to patient identity and clinical outcome.

### Cell lines and cell culture

The human thyroid cancer 8305C cell line was purchased from the European Collection of Cell Cultures (ECACC, Salisbury, United Kingdom). The thyroid carcinoma sw579 and human embryonic kidney 293T (HEK293T) cell lines were purchased from the American Type Culture Collection (ATCC, Manassas, VA, USA). The human thyroid carcinoma ARO, FRO, KAT5, and KAT10 cell lines were gifts from George G. Chen (The Chinese University of Hong Kong, Hong Kong SAR, China). All cells were cultured in DMEM supplemented with 10% fetal bovine serum (FBS, USA), penicillin (100 U/ml) and streptomycin (100 U/ml) at 37°C in a humidified 5% CO_2_ incubator.

### Antibodies and reagents

Mouse anti-E-cadherin and anti-vimentin antibodies were purchased from BD Technologies (La Jolla, CA, USA). Rabbit anti-ZO1 antibody was purchased from Invitrogen (Carlsbad, CA, USA). Rabbit anti-Snail antibodies, DMOG and LY294002 were purchased from Santa Cruz Biotechnology, Inc. (Santa Cruz, CA, USA). Antibodies against Slug, GSK3β, p-GSK3β (Ser9), Akt, p-Akt (Ser473), STAT3, and p-STAT3 (Thr705) were purchased from Cell Signaling Technology (Beverly, MA, USA). Rabbit anti-HIF-1α antibody was purchased from Abcam (Cambridge, MA, USA), and mouse anti-beta-actin antibody was obtained from Proteintech Group (Chicago, IL, USA). Goat anti-mouse (W4021) and goat anti-rabbit (W4011) peroxidase-conjugated secondary antibodies were obtained from Pierce Biotechnology (Rockford, IL, USA). The following reagents were used: anti-IL-11 neutralizing antibody (LifeSpan Technology, Newton, MA, USA), recombinant human IL-11 (rhIL-11) (Prospecbio, Rehovot, Israel), and Akt inhibitor V (Millipore, Billerica, MA, USA). All other reagents, unless otherwise specified, were purchased from Sigma-Aldrich (St. Louis, MO, USA).

### Generation of target-specific silenced cells

Retrovirus particles were produced in 293T cells by transient transfection of pSuper-retro plasmids harboring IL-11- and HIF-1α-specific RNAi target sequences. The RNAi target sequences were determined using Invitrogen's siRNA design tool (Invitrogen, Carlsbad, CA). The target sequences were as follows: IL-11 shRNA#1: 5′- GCATCTGTGCCTTATTTAT-3′; IL-11 shRNA#2: 5′- GCCTGGGCAGGAACATATA-3′; HIF-1α shRNA#1: 5′- GCTGATTTGTGAACCCATT-3′; HIF-1α shRNA#2: 5′- GCTGGAGACACAATCATAT-3′. A standard calcium phosphate co-transfection was performed with a PIK packaging plasmid in the 293T cells. After 24 hours, the transfection complexes were removed, and the cells were incubated in fresh DMEM (with 10% FBS) for an additional 24 hours. The supernatant was collected and filtered using 0.45-μm filters. The target (sw579) cells were incubated with the viral supernatant in the presence of 2 μg/ml Polybrene (Sigma-Aldrich). Twenty-four hours after infection, the cells were incubated with 1 μg/ml puromycin for 3 days for selection.

### Enzyme-linked immunosorbent assay (ELISA)

ATC cells were incubated in serum-free basal medium in the presence or absence of CoCl_2_ (0.1 mM). At the indicated periods, supernatant was collected and stored at −80°C until further use. IL-11 secretion in the cell culture supernatants was assayed using a human IL-11 Quantikine ELISA kit (R&D Systems, Minneapolis, MN, USA) according to the manufacturer's instructions. Total protein in the cells was estimated using the Bradford method.

### Western blotting

To analyze protein content in the cells, they were washed twice with PBS and then lysed in 1× sodium dodecyl sulfate (SDS) sample buffer. The protein concentration in the lysate was measured using a BCA protein assay. A total of 30 μg protein was separated on a 9% SDS-polyacrylamide gel by electrophoresis, transferred to a polyvinylidene fluoride (PVDF) membrane at 250 mA for three hours, blocked with 5% skim milk for one hour, and then incubated with primary antibodies at 4°C overnight, followed by incubation with secondary antibodies at room temperature for 50 min. After three washes, bound antibodies were visualized via electrochemiluminescence, which was captured using XAR film. Scanning and analysis of the western blot bands were performed using the Quantity One program (Bio-Rad, USA).

### Quantitative real-time polymerase chain reaction (quantitative RT-PCR)

Total RNA was isolated from the cells using TRIzol Reagent (Invitrogen, USA), and 2 μg of each sample was reverse-transcribed using M-MLV Reverse Transcriptase (Invitrogen, USA). Fast SYBR Green Master Mix was used to analyze the threshold cycle value of each sample by quantitative RT-PCR (Bio-Rad, USA). The housekeeping gene GAPDH was used as an internal control to normalize gene expression levels. The IL-11 sense primer was 5′-GCGGACAGGGAAGGGTTAAAG-3′, and the antisense primer was 5′-GGGCGACA GCTGTATCTGG-3′. The PCR amplifications were performed in a PTC-200 PCR system (Bio-Rad, USA) using the following cycle parameters: 10 min at 95°C, followed by 40 cycles of 10 s at 95°C, 10 s at 55°C, and 20 s at 72°C, with a final extension at 72°C for 10 min. The reactions were run in triplicate in three independent experiments.

### Cell invasion and migration assay

After being subjected to serum-starvation for 24 hours, 2×10^4^ sw579 cells or 4×10^4^ FRO cells were plated into the upper chambers (Corning Costar Corp., Cambridge, MA, USA) of transwell plates with or without a Matrigel coating (BD Biosciences, Bedford, MA, USA). Each of the lower chambers was filled with 500 μl DMEM supplemented with 10% fetal bovine serum. After incubation for 12 (migration) or 24 (invasion) hours, the cells that migrated to the reverse sides of the inserts were fixed in methanol for 10 min, stained with 0.5% crystal violet (Sigma-Aldrich) for 10 min, photographed, and counted (4 random 100× fields per well). Migrated cells were plotted as the average number of cells per field of view from 3 different experiments. The original magnification was 100×. Error bars represent SEM. *P < 0.05, **P < 0.01, ***P < 0.001.

### Wound healing assay

ATC cells were seeded in 6-well plates and grown to 95% confluence, after which they were serum-starved for 24 hours. Linear wounds were created with a pipette tip. The wounds were observed and photographed at various times, as indicated. The size of each wound was randomly measured at three sites. Each experiment was repeated at least three times.

### Cell proliferation assay

Cell proliferation was assessed using an MTT assay. First, the cells were seeded at a density of 1×10^3^ cells/well in triplicate in 96-well culture plates. The culture plates were then harvested at the indicated times post-seeding, and 20 μl of 5 mg/ml MTT was added to each well, followed by incubation for 4 hours. The culture medium was discarded, and 150 μl dimethyl sulfoxide (DMSO) was added to each well. Optical density (OD) was measured with a micro-culture plate reader (Bio-Rad, USA) at 490 nm. The absorbance values were normalized to the percentage of survival. Each experiment was performed in triplicate.

### Colony-formation assay

ATC cells were seeded at a density of 200 cells/well in triplicate in 6-well culture plates. The culture medium was changed every three days. After 10 days, the resulting colonies were fixed in methanol and stained with 0.5% crystal violet. Colonies containing more than 100 cells were counted.

### Statistical analysis

All statistical analyses were conducted using SPSS statistical package 19.0 (SPSS Inc., Chicago, IL, USA). The χ^2^ test for proportions was used to analyze association between IL-11 expression and the clinical features of the ATC patient cohort, as well as difference of IL-11 expression between the ATC tissues and PTC tissues. Student's t test was used for paired samples comparisons. *P* < 0.05 was considered statistically significant.

## SUPPLEMENTARY FIGURES


