# Supplementary material for: Interleukin-11 promotes epithelial-mesenchymal transition in anaplastic thyroid carcinoma cells through PI3K/Akt/GSK3β signaling pathway activation
Source: Oncotarget. 2016 Jul 25;7(37):59652–63. doi: 10.18632/oncotarget.10831 (PMC5312338; doi:10.18632/oncotarget.10831)
Supplement: Supplementary file 1 [file oncotarget-07-59652-s001.pdf]

Interleukin-11 promotes epithelial-mesenchymal transition in anaplastic thyroid carcinoma cells through PI3K/Akt/GSK3β signaling pathway activation

SUPPLEMENTARY FIGURES

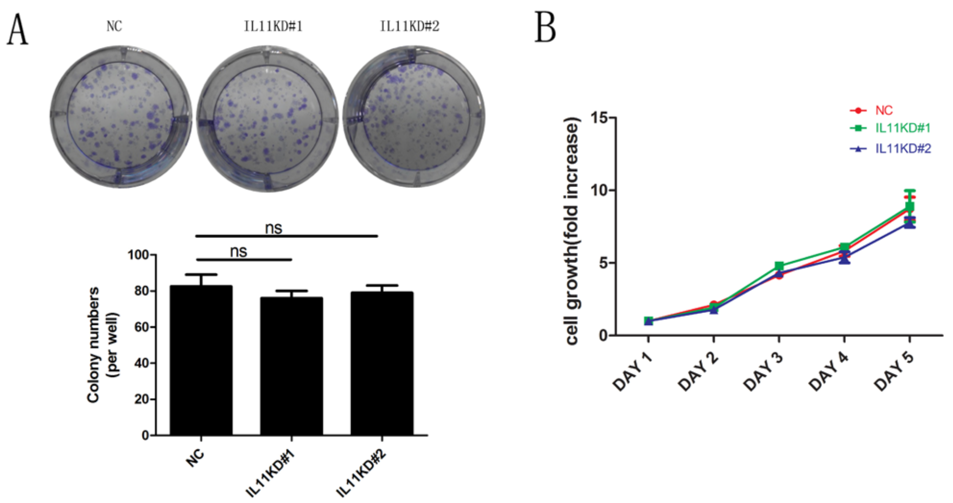

Supplementary Figure S1: Knockdown of IL-11 in sw579 cells does not influence cell proliferation. A. Colony formation assay or **B.** proliferation assay results for IL-11 shRNA- or NC-transfected sw579 cells.

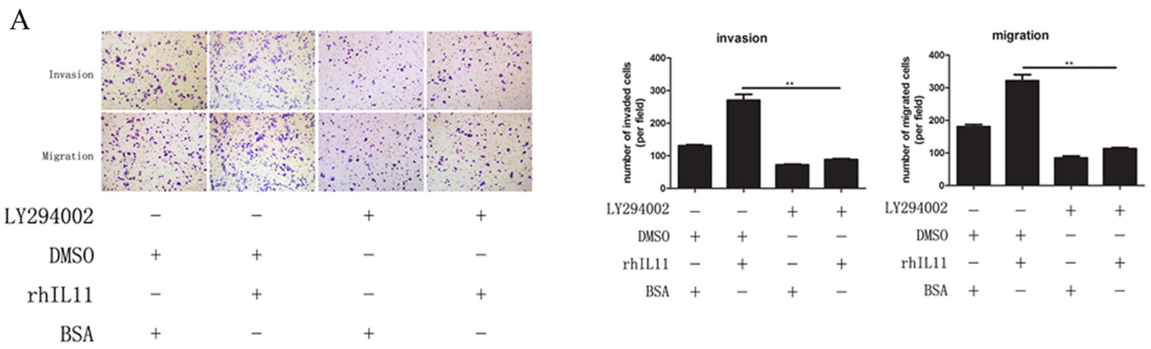

Supplementary Figure S2: IL-11 promotes the invasive and migratory abilities of ATC cells via the PI3K/Akt/GSK3β/Snail pathway. A. The invasive and migratory abilities of sw579 cells were evaluated by transwell assay after pretreatment with 20 μM LY294002 or 100 ng/ml rhIL-11. Migrated cells were plotted as the average number of cells per field. Photomicrographs are at 100× magnification. Bars correspond to the mean ± SD of three independent experiments.

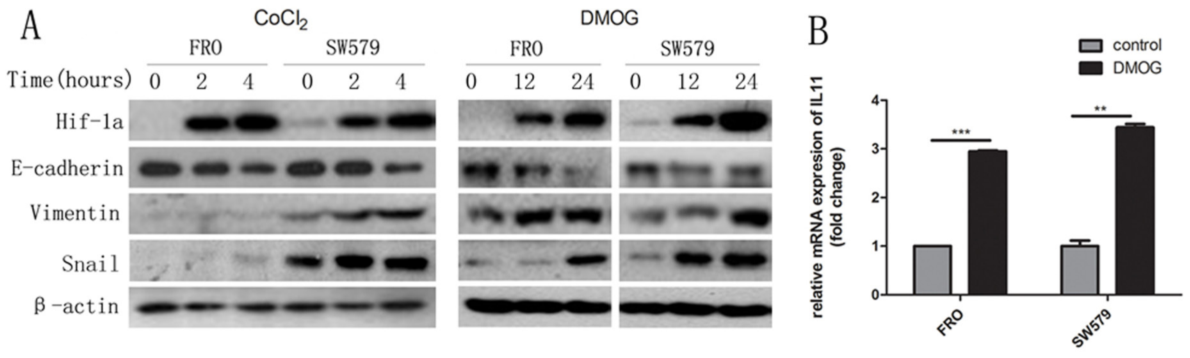

Supplementary Figure S3: Hypoxia induces EMT via HIF-1α. A. Western blotting of ATC cells after treatment with 0.1 mM $CoCl_2$ or 1 mM DMOG at the indicated time using the indicated antibodies. β-Actin was used as a loading control. **B.** IL-11 mRNA levels of FRO and sw579 cells treated with 1 mM DMOG.
